# Stephenson County Society of Physicians and Surgeons

**Published:** 1881-11

**Authors:** 


### ARTICLE XI.

### STEPHENSON COUNTY SOCIETY OF PHYSICIANS AND SURGEONS.

At a special meeting of this society which took place recently at the office of Dr. B. T. Buckley to take action concerning the death of Dr. L. A. Mease, the oldest practicing physician in Freeport, a graduate of Rush Medical College, of Chicago, and Jefferson Medical College, at Philadelphia, the following resolutions were unanimously adopted:

WHEREAS, It has pleased God to remove from among us, L. A. Mease, M.D., late President of this Society and for over thirty-five years a practicing physician in this county.

*Resolved*, That while we bow with humble submission to the Divine will, we cannot but regret the loss of a pioneer physician, whose death occurs at an age when we might reasonably hope to enjoy, yet for many years, the benefits of his ripe experience.

*Resolved*, That we recognized in him a courageous and skillful surgeon, fitted by anatomical and surgical knowledge and research, by judgment, coolness and manual dexterity to be, as he was for many years, the leading operative surgeon of this region; a competent and faithful physician ever ready to respond to the call of rich and poor alike, and to furnish to all the benefit of such skill and experience as, especially in the early days of his practice in this community, were as rare as they were precious to an infant settlement.

*Resolved*, That we tender to his bereaved family our sincere sympathy in their affliction.

*Resolved*, That a copy of these resolutions be spread upon the minutes and furnished the family of the deceased.

B. T. BUCKLEY,
F. W. HANCE,
LOUIS STOSKOPF,
*Committee.*

